# Evaluation of the dosimetry and centralization of scout-view function in CBCT

**DOI:** 10.1590/0103-6440202204926

**Published:** 2022-08-26

**Authors:** Danúsia da Silva Vilela, Luiz Roberto Coutinho Manhães, Monikelly do Carmo Chagas Nascimento, Anne Caroline Costa Oenning, José Luiz Cintra Junqueira, Elizabeth Ferreira Martinez

**Affiliations:** 1 Division of Oral Radiology, Faculdade São Leopoldo Mandic, Campinas, São Paulo, Brazil; 2 Division of Cell Biology, Faculdade São Leopoldo Mandic, Campinas, São Paulo, Brazil

**Keywords:** Scout-View, Cone-Beam Computed Tomography, Dosimetry, ROI

## Abstract

This study evaluated the centralization of the region of interest (ROI) in acquisition of the CBCT images, when the freely positionable scout-view (SV) function is applied. Additionally, the dosimetry of the acquired images was assessed in the SV function alone as well as in complete tomographic image in two different fields of view (FOV) (50x50 and 78x150mm). A three-location device was created to accommodate the dosimeters and the specimens, in the right, middle and left location during image acquisition. For dose assessment, thermoluminescent dosimeters were irradiated within the FOV and analyzed in a portable reader. For ROI evaluation, three specimens of gutta-percha stick were placed on the same device and the CT scans were acquired (CBCT OP 300 Maxio device, 90kV, 13mA, 85 µm voxel size, FOV of 50X50mm), with and without the SV, in three positions (3-9, 1-7 and 5-11 o’clock), simulating different regions of the mouth. Two image evaluations were performed, an objective and subjective. There was a slight percentage increase (1.36% to 1.40%) of the radiation dose with the use of SV. The distances were significantly greater in the images acquired without SV (p < 0.05). Every image obtained with SV was classified as being at the FOV’s center. In conclusion, the results demonstrated that SVs function is effective to centralize the ROI in the FOV, increasing the scan precision and avoiding repetitions due to positioning errors.

## Resumo

Este estudo avaliou a centralização da região de interesse (ROI) na aquisição das imagens de TCFC, quando a função scout-view (SV) posicionável livremente é aplicada. Adicionalmente, a dosimetria das imagens adquiridas foi avaliada isoladamente na presença da função SV, bem como após aquisição de imagem tomográfica completa em dois diferentes campos de visão (FOV) (50x50 e 78x150mm). Um dispositivo de três localizações foi criado para acomodar os dosímetros e os espécimes, na localização direita, central e esquerda, durante a aquisição das imagens. Para avaliação da dose, dosímetros termoluminescentes foram irradiados dentro dos campos de visão e analisados em leitor portátil. Para avaliação da ROI, três espécimes de guta percha foram colocados no mesmo aparelho e as tomografias foram adquiridas (CBCT OP 300 Maxio, 90kV, 13mA, 85 μm tamanho de voxel, FOV de 50X50mm), com e sem a SV, em três posições (3-9, 1-7 e 5-11 horas), simulando diferentes regiões da boca. Foram realizadas duas avaliações de imagem, uma objetiva e outra subjetiva. Houve um leve aumento percentual (1,36% para 1,40%) da dose de radiação com o uso de SV. As distâncias foram significativamente maiores nas imagens adquiridas sem SV (p < 0,05). Todas as imagens obtidas com SV foram classificadas como sendo do centro do FOV. Em conclusão, os resultados do presente estudo demonstraram que a função scout view é eficaz para centralizar a ROI no FOV, aumentando a precisão do escaneamento e evitando repetições devido a erros de posicionamento.

## Introduction

The radiation dose in cone beam computed tomography (CBCT) scans is causing a growing concern due to the widespread use in Dentistry and due to the stochastic biological effects. The field of view (FOV) choice is made taking into account the region of interest (ROI) and the clinical needs of each patient ^1^ ^,^ ^2^. In addition, the choice of a FOV restrict to the ROI reduces the effective dose and minimizes the scattering radiation, improving the image quality, thereby respecting ALADA principle ("as low as diagnostically acceptable") ^3^.

Considering the use of CBCT in Pediatric Dentistry, the European project DIMITRA (Dentomaxillofacial Pediatric Imaging: an Investigation Towards Low-Dose Radiation Induced Risks) ^(^ ^4^ proposed the evolution of the ALARA ("as low as reasonably achievable") and ALADA principles to ALADAIP (As Low as Diagnostically Acceptable being Indication-oriented and Patient-specific). DIMITRA proposes an examination with the best image quality accompanied by the lowest possible radiation dose based upon the indication and individuality of each patient ^5^ ^,^ ^6^.

In this context, the operator's lack of experience in positioning the patient in the X-ray device, the inadequate adjustment of the FOV, or any physical movement of the patient during image acquisition can result in the need for the CBCT to be repeated, thereby doubling the patient´s radiation dose ^7^. Fortunately, the most current devices of a CBCT have the scout-view (SV) function which gives the ability to certify whether the selected FOV ensures that the entire ROI will be included prior to the image´s final exposure ^8^.

The SV function involves a low-dose of radiation to acquire a preview image of the ROI. Despite of this very low exposure, the SV is considered an extra dose of radiation. The SV, however, allows a preview of the correct and effective positioning of the ROI within the FOV, thereby avoiding an exam re-taken with a consequential improvement in the patient’s protection ^9^ ^,^ ^10^ ^,^ ^11^ ^,^ ^12^. Considering the linear non threshold hypothesis (LNTH) as the current more accepted model to manage the radiation risk, even low doses might trigger the stochastic effects like cancer. Therefore, this study aimed to oppose the dosimetry of the SV function to the centralization assessment of the ROI in CBCT scans, in order to investigate the practical value of the SV function.

## Material and methods

### Sample groups and device used

For dosimetry analysis, a total of 25 thermoluminescence dosimeters (TL-LiF) were used. One dosimeter was placed outside of the examination room in order to measure the average background radiation dose.

For evaluation of the object positioning in the ROI related to the center of the FOV, a specimen of gutta-percha stick (Odahcam, Dentisply, Brazil) was used, considering the smallest image artifact for this material ^13^.

**Figure 1:**
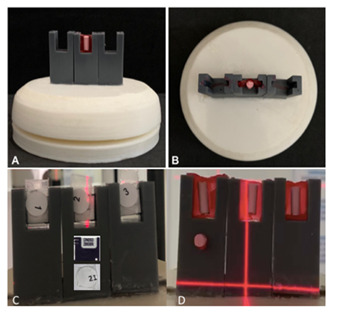
Device used for positioning of dosimeters and specimens. (A) and (B) show the positioning of a specimen in the middle of the device, in the front view (A) and top view (B). (C) Positioning of the dosimeters in the right ^1^, middle ^2^ and left ^3^ location. (D) Positioning at the right side of a small piece of gutta-percha on the front of the device.

A device of acrylonitrile butadiene styrene (ABS, Done 3D, Ribeirão Preto, Brazil) containing three locations was made to accommodate the dosimeter and, after the specimens in the right ^1^, middle ^2^ and left ^3^ location (Figure 1 A and B).

The scout-view and CBCT acquisitions were performed using CBCT OP 300 Maxio device (Instrumentarium, Helsinki, Finland), according the manufacturer’s recommendation using the standard protocol (90kV, 13mA). The time for each shot for CBCT acquisition was 2,34s and 4,50s, for FOV 50X50 mm and 78X150mm, respectively. For each shot for scout view acquisition, the time was 0,02s and 0,04s, for FOV 50 X 50 mm and 78X150mm, respectively.

### Dosimetry

The assessment was performed on FOV 50 X 50 mm and FOV 78 X 150 mm. In order to organize image acquisitions and data tabulation, four groups were determined based on the isolated presence of SV or associated with FOV. These groups were named G1, G2, G3 and G4, as described in Table 1.

**Table 1:** Distribution of sample groups for dosimetry analysis.

Groups	Description
G1 (n=6)	Isolated SV (FOV 50X50 mm)
G2 (n=6)	SV+ FOV acquisition 50X50 mm
G3 (n=6)	Isolated SV (FOV 78X150 mm)
G4 (n=6)	SV+ FOV acquisition 78X150 mm

Dosimetry was performed in the SV alone (15 shots; G1 and G3), as well as in complete tomographic image (SV + FOV, 5 shots; G2 and G4), at 50 X 50 mm and 78 X 150 mm FOV. The number of the shots to achieve measurable radiation values was carried out according to a pilot study.

Dosimeter readings were taken after exposures using a reader (Landauer^TM^ microSTARii^TM^, Vélizy-Villacoublay Cedex, France).

The acquisitions were carried out in duplicate to ensure the accuracy of the experiments.

### Evaluation of the specimen in relation to the FOV’s center

The specimens (gutta-percha) were positioned on the device in three locations and were named object 1 (right), object 2 (middle) and object 3 (left). To identify the positioning of the specimen, a small piece of gutta-percha (3mm length) was placed on the front of the device, at the right side (Figure 1 C and D).

The FOV elected to calculate the positioning of the specimen, as it relates to the center of the FOV, was 50 X 50mm because this is the smallest one in this CBCT unit (presenting the greatest difficulty in positioning). This assessment was made with and without the SV function.

**Figure 2:**
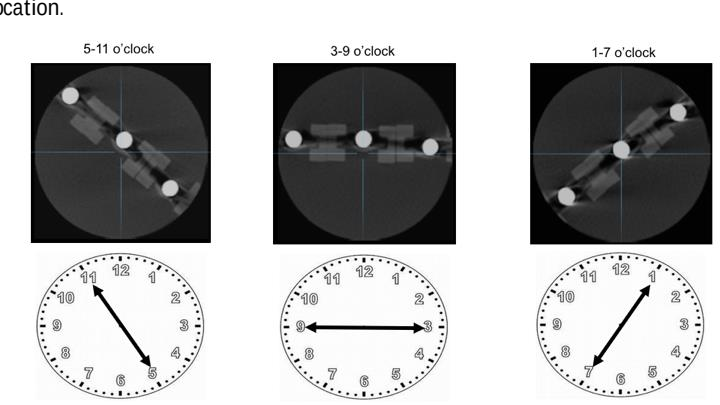
Specimens placed in the positions of 5-11o’clock, 3-9 o’clock and 1-7 o’clock. FOV’s center represented by the intersection of the reference lines.

Three image acquisitions were performed centralizing the objects within the ROI, with and without the SV, in three different positions named as 9 o’clock, 1-7 o’clock, 5-11 o’clock; each position simulating different locations within the mouth (Figure 2). The repetitions were performed interspersed and randomly, in order to repeat the position of the specimen within the FOV’s center. For the acquisition of images without the SV, the light guides of the devices were used to center the specimen within the desired location.

The tomographic images of the specimens were assessed in three different days and was objectively measured and subjectively evaluated by two trained and experienced observers.

**Figure 3:**
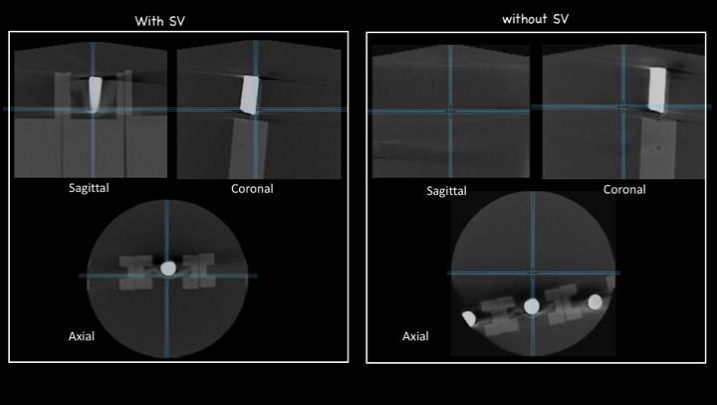
Multiplanar reconstruction window. The intersection of the lines is in the center of the FOV at the sagittal, coronal and axial sections.

**Figure 4:**
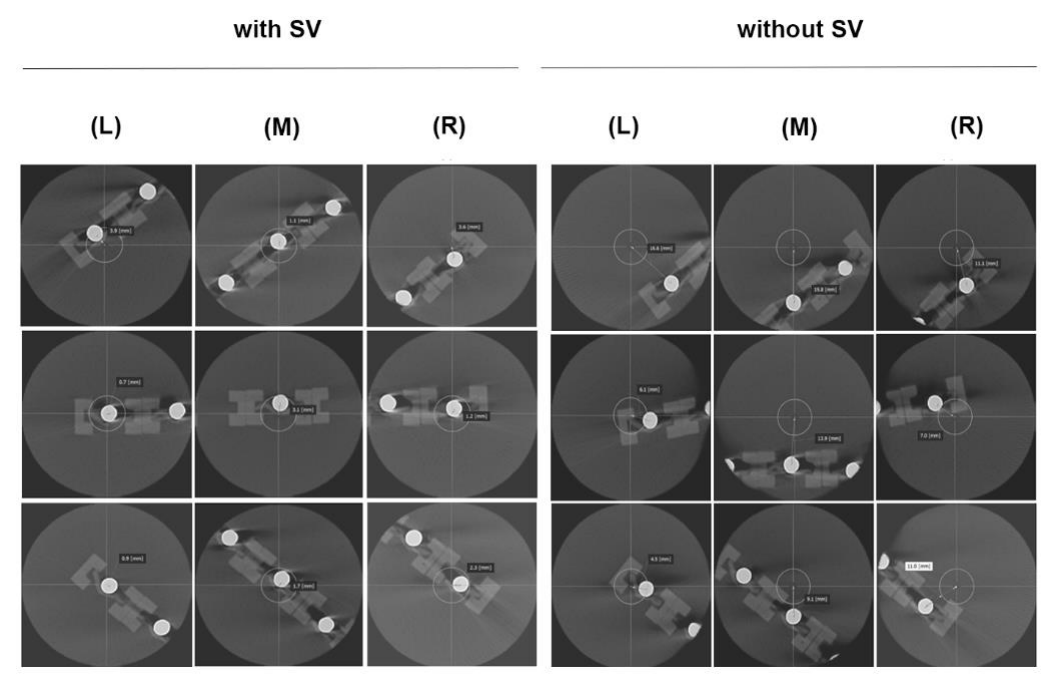
Measurements taken from the center of the specimen to the center of the FOV with and without SV at (L) left, (M) middle and (R) right positions.

In order to standardize the objective (quantitative) evaluation at the Xelis^TM^ dental viewer software (LED dental, USA), the center of the FOV and the lower part of the specimen were located in the sections (axial, sagittal and coronal) of the multiplanar reconstruction window (Figure 3). In the axial reconstruction, distance measurements were made from the specimen to the FOV’s center, indicated by the intersection of the reference lines (Figure 4). Only the brightness and contrast could be adjusted by the observers; the use of improvement filters was not allowed.

**Figure 5:**
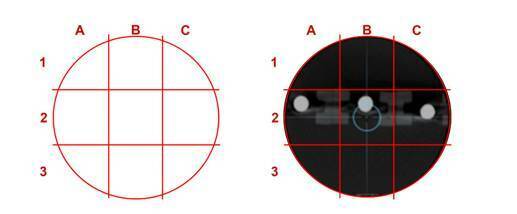
Grid used to evaluate the positioning of the specimens in relation to the center of the FOV.

A score was applied for the subjective (observational) assessment. A circular grid (50 mm of diameter) was designed and sectioned by four lines, creating 3 sections on the X-axis (1, 2 and 3) and 3 sections on the Y-axis (A, B and C) (Figure 5). This grid was positioned over the axial section to check where the specimen was located within the FOV. Position 2B was identified as the zone within the center of the FOV and considered as the ideal location for acquisition.

### Statistical analysis

For a comparative analysis of the dosimetry images, after verifying the normalization of the data, a *Student* t-test was applied. For the evaluation data of the specimen in relation to the FOV’s center (with and without SV), it was calculated the averages of the distances of the three measurements performed on the same images. Then, a descriptive and exploratory analyzes of the data was performed. For the variable location of the FOV (in the center or outside the center), it was calculated the mode (highest frequency) of the three evaluations that were performed on the same images. The chi-squared test was then applied. All analyzes were performed on the R program, and a 5% significance level was considered.

## Results

### Dosimetry

Table 2 shows that for the FOV 50X50mm, the average values obtained for the absorbed dose were 4.68 (±0.71) mSV for the acquisition of the complete image and 0.064 (±0.012) mSV, for the SV only. For the FOV 78X150mm, it was observed that the average value obtained for the dose was 5.03 (±1.29) mSV for the acquisition of the complete image and 0.07 (±0.02) mSV, for the SV only.

The percentage gain value was 1.36% and 1.40%, respectively, for the FOV 50X50mm and 78X150mm. Therefore, regardless of the size of the FOV as well the use of SV function, the radiation dose was statistically the same (p > 0.05).

**Table 2:** Dosimetry obtained in the different groups. Mean values (standard deviation), expressed in mSV, and percentage differences, representing dose gain for each size of FOV.

Groups	mSV	%
G1	0.064 (±0.012)	1.36
G2	4.68 (±0.71)	
G3	0.07 (±0.02)	1.4
G4	5.03 (±1.29)	

Caption: G1 = SV only (FOV 50X50 mm), G2 = SV + FOV 50X50 mm, G3 = SV only (FOV 78X150 mm), G4 = SV + FOV 78X150mm.

### Evaluation of the specimen in relation to the FOV’s center

**Figure 6:**
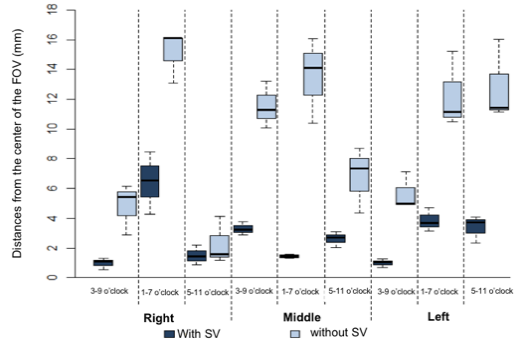
Box-plot from the FOV’s center according to the use of the SV function and position.

Table 3 and Figure 6 show the results of the distances from the center of the FOV. There was a significant difference between the distances measured with and without SV (p < 0.05) in all evaluated positions. It can be noted that the distances were significantly greatest in the images taken without the SV function (p < 0.05). In both studied conditions (with and without SV function), there was a significant difference between the positions (p < 0.05).

**Table 3:** Mean (standard deviation), in mm, from the FOV’s center according to the use of the SV function and position.

Position		With SV	Without SV
Right	3-9 o’clock	0.99 (±0.38) Bc	4.82 (±1.70) Ab
	1-7 o’clock	6.44 (±2.10) Ba	15.11 (±1.77) Aa
	5-11 o’clock	1.50 (±0.67) Bb	2.30 (±1.60) Ac
Middle	3-9 o’clock	*^$^3.30 (±0.44) Ba	*^$^11.52 (±1.58) Aa
	1-7 o’clock	*^$^1.44 (±0.15) Bb	13.52 (±2.88) Aa
	5-11 o’clock	*2.61 (±0.54) Ba	*^$^6.08 (±2.21) Ab
Left	3-9 o’clock	1.00 (±0.29) Bb	5.68 (±1.26) Ab
	1-7 o’clock	*3.86 (±0.78) Ba	12.28 (±2.58) Aa
	5-11 o’clock	*3.39 (±0.93) Ba	*12.87 (±2.74) Aa

Caption: *Differs from the right position under the same conditions as the other factors (p <0.05). ^$^Differs from the left position under the same conditions as the other factors (p <0.05). Different letters (upper case comparing horizontally between with and without scout-view and lower case comparing vertically between each position under the same conditions as the other factors) indicate significant differences (p <0.05).

Table 4 shows a significant association between the use of the SV function and the location of the object image inside the FOV, by subjective analysis (p < 0.05). All object images acquired with the SV function were classified as being in the center of the FOV (position 2B). Meanwhile, among the images acquired without the SV, 55.6% were classified as the object out of the center.

**Table 4:** Absolute and relative location in the FOV by subjective evaluation according to the use of the SV function and position.

Position		With SV		Without SV	
		Centered	Out of Center	Centered	Out of Center
Right	3-9 o’clock	3 (100.0%)	0 (0.0%)	3 (100.0%)	0 (0.0%)
	1-7 o’clock	3 (100.0%)	0 (0.0%)	0 (0.0%)	3 (100.0%)
	5-11 o’clock	3 (100.0%)	0 (0.0%)	3 (100.0%)	0 (0.0%)
Middle	3-9 o’clock	3 (100.0%)	0 (0.0%)	0 (0.0%)	3 (100.0%)
	1-7 o’clock	3 (100.0%)	0 (0.0%)	0 (0.0%)	3 (100.0%)
	5-11 o’clock	3 (100.0%)	0 (0.0%)	3 (100.0%)	0 (0.0%)
Left	3-9 o’clock	3 (100.0%)	0 (0.0%)	3 (100.0%)	0 (0.0%)
	1-7 o’clock	3 (100.0%)	0 (0.0%)	0 (0.0%)	3 (100.0%)
	5-11 o’clock	3 (100.0%)	0 (0.0%)	0 (0.0%)	3 (100.0%)
Total Images		27 (100.0%)*	0 (0.0%)**	12 (44.4%)*	15 (55.6%)**

Caption: * and ** indicates statistical difference (p <0.05)

## Discussion

The results of the present study revealed that prior to image acquisition, the scout preview function proves to be a useful tool for screen alignment, being effective to centralize the ROI in the FOV, increasing the scan precision and avoiding repetitions due to positioning errors.

The biological effects from the radiation exposures and the high demand for ordering CBCT exams in the dental field has raised concerns about the dose received by patients. In this sense, the principle of justification must be respected in the indications for radiographic examinations. Additionally, considering the greater radiosensitivity of the children, imaging requests should be guided by the appropriate indications, being specific for each patient following the ALADAIP principle ^3^ ^,^ ^4^ ^,^ ^5^ ^,^ ^6^ ^,^ ^14^ ^,^ ^15^.

Because of this, dental surgeons require constant reminding of these radioprotection measures and the biosafety protocols must be respected ^16^. In this scenario, it is important to emphasize that most of the CBCT units have the SV function thus providing a means to ascertain whether the ROI is included in the selected FOV prior to the exposure, thus avoiding repetition of exams ^8^.

Despite the advantage of small FOVs on radiation dose reduction, a higher number of repetitions compared to large ones may occur, leading to re-exposures without any additional benefits ^8^ ^,^ ^17^. In this context, despite the additional low dose exposure of the scout view, it may be considered a radioprotection tool given that it avoids re-exposures for positioning errors.

Despite the well-known low dose involved in the SV exposure, there is a lack of studies and scientific data on the dosimetry of the SV. Thus, the present study evaluated the dosimetry of two FOV sizes, 50X50 mm and 78X150 mm, in one CBCT unit (OP 300 Maxio), opposing this information to the assertiveness for obtaining images of the ROI within the center of the FOV. If the structure to be analyzed, the ROI, is eccentric in the field of view, the image evaluation might be jeopardized by truncation artifacts ^5^. Regarding the possible extra dose of ionizing radiation with the use of SV, confirmed as low by the findings of the present study, the protection offered to the patient is evident, since eventual double-exposure is avoided for positioning error ^7^ ^,^ ^8^ ^,^ ^9^.

Smaller FOV sizes hinders the correct positioning of the ROI within its center and causes greater difficulty for the operator, thus increasing the chance of repeating the exam ^17^. Additionally, in most of the times, CBCT exams are repeated the lack of experience of the operator ^18^, which reinforces the importance of the use of SV before exam acquisition.

 In this study the small FOV option of the OP300 Maxio (50 X 50 mm) was tested. Although not all CBCT units have the SV, the devices have guide lights, which assist in positioning the patient within the FOV’s area previously to the acquisition, in accordance with the Frankfurt and mid-sagittal plans ^11^ ^,^ ^12^. The present study reinforces the importance of using the SV, that lies in the fact that the chances of an error of centralization of the ROI increases when using only the guide lights. This risk of error also increases when associated with the lack of operator experience ^7^ ^,^ ^11^ ^,^ ^12^.

For the analysis of the ROI in relation to the center of the FOV, the results showed that there was a significant difference between the distances measured with and without SV in all of the evaluated positions. It was noted that the distances were significantly greater in the images taken without the SV function (p <0.05). It was found that 55.6% of the images obtained without the SV were displaced away from the center of the FOV. In contrast, all images obtained with the SV were classified as being in the center of the FOV.

The results obtained in the present study reinforce the importance of the SV for positioning the ROI. This finding is especially important using smallest FOV (50 X 50 mm), in which in addition to the difficulty of positioning the patient in the device by the operator ^6^ ^,^ ^7^ ^,^ ^8^ ^,^ ^9^ ^,^ ^19^, any movement during the scan may lead to an extra exposure of the patient, due to the exam repetition ^16^ ^,^ ^20^.

However, considering that this study assessed an *in vitro* analysis, future studies should be performed in order to reproduce the proposed methodology in dosimetric phantoms or skulls. Additionally, other CBCT equipments, with different exposure geometries, and different FOV sizes and morphologies could be tested.

Therefore, despite the low dose of radiation offered by SV, the results highlighted its importance as a radioprotection tool. All obtained images with the SV, allowed to position the ROI in the center of the FOV, increasing the scan precision which allows to avoid positioning error and, in consequence, re-exposure of the patient.
